# Can Resveratrol-Inhaled Formulations Be Considered Potential Adjunct Treatments for COVID-19?

**DOI:** 10.3389/fimmu.2021.670955

**Published:** 2021-05-19

**Authors:** Giovanni A. Rossi, Oliviero Sacco, Antonino Capizzi, Paola Mastromarino

**Affiliations:** ^1^Department of Pediatrics, Pediatric Pulmonary Disease Unit, IRCCS Istituto Giannina Gaslini, Genoa, Italy; ^2^Department of Public Health and Infectious Diseases, Microbiology Section, University of Rome “Sapienza”, Rome, Italy

**Keywords:** COVID-19, resveratrol, antiviral, anti-inflammatory, inhaled formulations

## Abstract

The pandemic caused by severe acute respiratory syndrome coronavirus type 2 (SARS-CoV-2) has led to an extraordinary threat to the global healthcare system. This infection disease, named COVID-19, is characterized by a wide clinical spectrum, ranging from asymptomatic or mild upper respiratory tract illness to severe viral pneumonia with fulminant cytokine storm, which leads to respiratory failure. To improve patient outcomes, both the inhibition of viral replication and of the unwarranted excessive inflammatory response are crucial. Since no specific antiviral drug has been proven effective for the treatment of patients and the only upcoming promising agents are monoclonal antibodies, inexpensive, safe, and widely available treatments are urgently needed. A potential anti-inflammatory molecule to be evaluated, which possesses antiviral activities in several experimental models, is the polyphenol resveratrol. This compound has been shown to inhibit SARS-CoV-2 replication in human primary bronchial epithelial cell cultures and to downregulate several pathogenetic mechanisms involved in COVID-19 severity. The use of resveratrol in clinical practice is limited by the low bioavailability following oral administration, due to the pharmacokinetic and metabolic characteristics of the molecule. Therefore, topical administration through inhaled formulations could allow us to achieve sufficiently high concentrations of the compound in the airways, the entry route of SARS-CoV-2.

## Highlights

Relatively inexpensive, nontoxic, and widely available treatments effective against COVID-19 are urgently needed. A potential anti-inflammatory molecule that possesses antiviral activities in several experimental models is the polyphenol resveratrol. This compound inhibits SARS-CoV-2 replication in primary bronchial epithelial cell cultures and downregulates several pathogenetic mechanisms involved in COVID-19 severity. Due to the low bioavailability following oral administration, topical administration of the resveratrol through inhaled formulations could allow to achieve sufficiently high concentrations of the compound in the airways and to limit SARS-CoV-2 replication in COVID-19 patients.

## Introduction

The outbreak of the novel coronavirus SARS-Cov-2 first occurred in Wuhan, Hubei, China, in December 2019, and, on March 11, 2020, the SARS-Cov-2-induced disease, named COVID-19, was declared a global pandemic (1). The rapid spread of the virus put health care systems under pressure and forced much of the world to adopt lockdown strategies to contain the growth of COVID-19. As compared to the previous 2002–2003 SARS-CoV outbreak, SARS-CoV-2 infection is characterized by lower pathogenicity and mortality rates but appears to be much more contagious and able to induce a wider clinical spectrum, ranging from asymptomatic infection to severe viral pneumonia, acute respiratory distress syndrome (ARDS), septic shock and multiple organ failure (2, 3). The severity of COVID-19 is a consequence of lung inflammation and injury caused by the viral infection but also of an overstated inflammatory reaction, related to the inefficiency of immune response triggered to control the virus: the cytokine storm (1, 4). Despite the unceasing increase in studies, there is currently no proved efficacy for any therapeutic approach for COVID-19 patients (5–7). Relatively inexpensive, nontoxic, and widely available treatments are therefore urgently needed. which, in addition to inhibiting viral replication, could modulate some important consequences of COVID-19 infection, such as the cytokine storm (4). A potential anti-inflammatory molecule to evaluate, which possesses these activities in several experimental models, is the natural compound resveratrol (trans-3,5,4’-trihydroxystilbene) (8–11). The aim of this manuscript was to present an overview of the antiviral and anti-inflammatory properties of resveratrol with a focus on SARS-Cov-2. The administration modes and routes to be used to overcome the pharmacokinetic and metabolic limitations that characterize this molecule will also be summarized, arguing how the topical administration to the airways through inhaled formulations could be an option to be considered and evaluated. We undertook electronic searches in the Giannina Gaslini Institute living search database, which is updated daily with published articles from PubMed and Embase and with preprints from medRxiv and bioRxiv. We did not apply any language restrictions.

## The Natural Polyphenolic Compound Resveratrol

Resveratrol is a natural polyphenolic compound produced by several plants in response to physiologic stress or to bacterial and fungal infections (12). This molecule shares multiple bioactivities beneficial for human health, possessing antioxidant, anti-tumor, and antiviral properties (12). Much of the research in the last 10 years has focused on effects on diseases related to type 2 diabetes; cardiovascular diseases; neurological diseases; and breast, colorectal, liver, pancreatic, and prostate cancers (12, 13). However, resveratrol has been reported to exhibit also antiviral properties against the most common respiratory viruses through multiple cellular targets and pathways (8, 14, 15).

## The Anti-Viral Activity of Resveratrol

**Figure 1:**
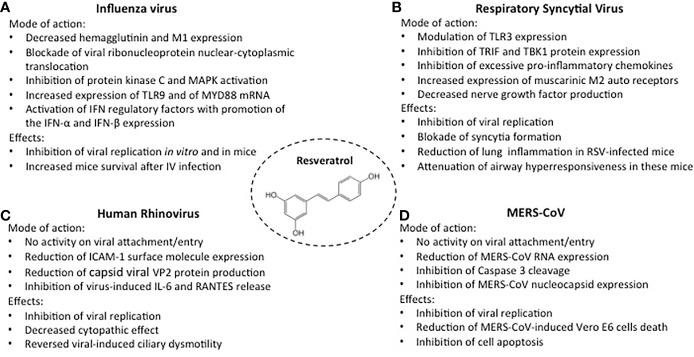
Activities of resveratrol against the most common respiratory viruses. Mode of action and inhibitory effects on Influenza virus, (**A**); Respiratory Syncytial virus, (**B**); Human Rhinovirus, (**C**); MERS-CoV, (**D**).

Resveratrol can interfere with viral replication through inhibition of viral gene expression, viral nucleic acid, and protein synthesis and through downregulation of various cellular transcription and signaling pathways(8, 16–19). These anti-viral activities can also dampen the viral-induced excessive inflammatory response and limit the associated lung parenchyma injury (20, 21). These properties have been demonstrated in infections induced by the influenza virus, respiratory syncytial virus, human rhinovirus, and middle east respiratory syndrome coronavirus (**Figure 1**) (8, 14, 16).

### Influenza Virus

Influenza virus infection is characterized by significant epithelial cell damage and a disease severity proportionate to the magnitude of the viral cytotoxic capacity (22). The ability of resveratrol to inhibit influenza virus A/Puerto Rico/8/34 H1N1 virus was tested in MDCK cell cultures (23). Resveratrol, at concentrations of 10-20 mg/mL did not induce significant cytotoxic effects in uninfected cells but reduced viral replication, an effect remaining stable through 72 hours after infection (**Figure 1A**). The antiviral effects did not involve the inactivation of the virus, the inhibition of viral adsorption, or the prevention of virus entry into the host cells. Indeed, resveratrol pretreatment of the virus or of the uninfected cells, or the presence of resveratrol in cell cultures during the phase of viral adsorption, did not modify susceptibility to infection (23). In contrast, maximum inhibition of viral replication was achieved when resveratrol treatment began 3 hours after virus challenge, but no effects were detectable when the drug was added 9 hours after infection. Thus, the antiviral activity was likely related to posttranscriptional events. Indeed, the inhibition of matrix protein 1 (M1), hemagglutinins (HA0, HA1, and HA2), and neuraminidase expression was linked to blockade of nuclear-cytoplasmic translocation of viral ribonucleoproteins and to downregulation of protein kinase C (PKC) and mitogen-activated protein kinase (MAPK) activity and their dependent pathways.

Moreover, in four-week-old female BALB/c mice resveratrol improved survival and decreased influenza viral titers in the lungs (23). Similar *in vitro* results were obtained in A549 cell cultures: resveratrol 25 mg/mL inhibited the replication of clinically isolated strains of H1N1 influenza virus, downregulating hemagglutinin and neuraminidase expression (24). Inhibition of H1N1 replication was associated with modulation of the host-cell immune response through increased expression of Toll-like receptor (TLR)9 and of MYD88 mRNA, a signal transduction adaptor involved in TLR9 activation. TLR9 promotes the expression of IFN-α and IFN-β, cytokines involved in the first lines of defense against viral infections (25). Lastly, resveratrol 25 mg/mL was also able to inhibit the infection of the human influenza B virus in monolayers of MDCK cells and Vero cells (26).

### Respiratory Syncytial Virus (RSV)

Besides the direct cytopathic effect of the virus, the severity of RSV disease in infants appears to be provoked by a sustained, ineffective inflammation induced by a deficient, immature immune response (27, 28). Resveratrol can both affect RSV replication and modulate the viral-induced inflammatory response. In human tracheal epithelial cell line cultures, infected with human RSV A2-strain, resveratrol significantly lowered RSV titers at 24-, 48- and 72-hour incubation and blocked syncytial formation (**Figure 1B**) (29). The inhibitory activity was associated with decreased IL-6 production, related to a dampening effect on the expression of TIR-domain-containing adapter inducing IFN-β (TRIF) and TANK binding kinase 1 (TBK1) protein. IL-6 is an essential cytokine that transmits defense signals following a pathogen invasion, but its excessive and sustained production is associated with disease severity (30). Uncontrolled response to RSV can be mediated by excessive activation of TLR3, which may contribute to disease worsening (31). In immunocompromised BALB/c mice infected with human RSV A2-strain, TLR3 activation stimulated an excessive IFN-g induction that, in this animal model, is associated with severe airway hyperresponsiveness (32, 33). In these mice, resveratrol treatment modulated TLR3 expression and significantly reduced the RSV lung titers, the number of infiltrating lymphocytes in bronchoalveolar lavage fluid (BALF), and the recruitment of leukocytes into lung tissues (32, 33). Furthermore, following RSV infection, resveratrol attenuated airway hyperresponsiveness to methacholine, significantly decreasing BALF IFN-g levels, and promoting the expression of autoinhibitory muscarinic M2 receptors (33). Through a negative feedback mechanism, M2 muscarinic receptors can inhibit the release of acetylcholine, a neurotransmitter that promotes airway contraction. In a subsequent study in the same experimental model, resveratrol-induced suppression of persistent airway inflammation and airway hyperresponsiveness was detectable 60 days after RSV infection and was associated with decreased levels of BALF nerve growth factor (NGF) (14, 34). In addition to being a neurotrophic factor and inhibiting infected cell apoptosis, NGF acts in RSV infection as a promoter of acetylcholine release and as a signaling molecule inducing neuropeptide production and neurogenic inflammation (35).

### Human Rhinovirus (HRV)

Epithelial cell cytotoxicity does not appear to play a major role in HRV-induced disease, which is instead characterized by the production of mediators of inflammation by infected structural and inflammatory cells (14, 36). In addition to releasing large amounts of IL-6 and RANTES, following HRV infection, airway epithelial cells upregulate the expression of intercellular adhesion molecule-1 (ICAM-1), the major surface cellular receptor for HRV A and B (37–39). In a study on human nasal epithelia exposed to HRV-serotype 16, resveratrol 75-300 mM exhibited a dose-dependent activity against virus replication, not related to inactivation of extracellular viral particles or to inhibition of viral attachment/entry (**Figure 1C**) (40). A progressive decrease of ICAM-1 expression was observed, associated with a slight reduction of its molecular weight, likely related to a lower degree of ICAM-I glycosylation, a process that has been shown to negatively affect HRV binding (41). Moreover, even at the lowest concentration, resveratrol dramatically reduced the levels of the structural capsid viral proteins VP2, indicating a high efficacy against HRV-16 viral protein synthesis. Finally, resveratrol reversed the reduction of ciliary motility and the increased IL-6, IL-8, and RANTES release induced by the HRV-16 infection (40).

### Middle East Respiratory Syndrome Coronavirus (MERS-CoV)

MERS-CoV is the agent responsible for a respiratory infection firstly identified in June 2012 in Jeddah, Saudi Arabia (42). In the early phase of the infection, MERS-CoV caused relevant cell cytotoxicity and a severe inflammatory response (43). Studies performed on a Vero E6 cell line showed that resveratrol 125-250-μM, significantly inhibited MERS-CoV infection and reduced cytotoxicity and cell death (**Figure 1D**) (44). Dose-dependent inhibition of Caspase 3 cleavage was observed, i.e., of the protein whose sequential activation plays a central role in the execution phase of cell apoptosis (45). At 48 hours post-infection, the levels of MERS-CoV RNA and of MERS-CoV infectious titers were significantly lowered in resveratrol-treated cells, an inhibitory effect also detectable when resveratrol was added to the cell cultures at 3 hours post-infection, suggesting that the inhibitor effect occurs after viral adhesion and entry. Relatively high concentrations are needed to deliver antiviral effects since the inhibition of the expression of MERS-CoV nucleocapsid (N), a protein essential for MERS-CoV replication was observed only when the cell cultures were exposed to resveratrol 250 μM (44). However, the addition every 24 hrs of a significantly lower compound concentration (62.5 μM) inhibited viral titers by approximately 10 folds and partially rescued MERS-Cov-induced cell death.

### Severe Acute Respiratory Syndrome Coronavirus 2 (SARS-CoV-2)

At the whole-genome level, SARS-CoV-2 shares a 50% sequence identity with MERS-CoV, but the presence of amino acid substitutions in the structural proteins explains the functional differences between the two coronaviruses and, possibly, the different response to anti-viral agents (46). As compared with MERS-CoV, SARS-CoV-2 infection appears to much less lethal but much more contagious, rapidly spreading to all continents (47).

## COVID-19 Pathogenesis and the Cytokine Storm

SARS-CoV-2 targets airway epithelial cells through the structural spike protein which contains a receptor-binding domain specifically recognizing angiotensin-converting enzyme 2 (ACE2) as its receptor on host cells (3). To enter host cells, coronaviruses first bind to a cell surface receptor for viral attachment, subsequently enter endosomes, and eventually fuse viral and lysosomal membranes. In addition to receptor binding, protease activators (cell surface protease TMPRSS2 and lysosomal proteases) are important for SARS-CoV-2 entry (3, 48).

At the alveolar level, infection of type I and II pneumocytes and of capillary endothelial cells is followed by the release of pro-inflammatory cytokines, recruitment, and activation of inflammatory and immune-effector cells (49). The persistent, exaggerated inflammatory response and the activation of the kinin-kallikrein system result in increased alveolar vessel permeability, vascular leakage leading to angioedema and hyaline membrane formation whilst the activation of coagulation leads to intravascular microthrombus formation (49).

## Inhibition of SARS-CoV-2 Replication by Resveratrol

The ability of resveratrol to inhibit SARS-CoV-2 replication was evaluated in Vero cells by a quantitative reverse transcription-polymerase chain reaction and immunofluorescence assay (50). The results showed that resveratrol significantly inhibited dose-dependently the replication of SARS-CoV-2 with an EC50 (half-maximal effective concentration) of 4.48 μM. Time of addition assay demonstrated that SARS-CoV-2 replication, in multiple cycles viral replication conditions, was inhibited by 98% when 50 μM resveratrol was added to cells after the virus adsorption step while treatment of cells with the compound for 2 hr before virus infection produced a very low inhibition. Interestingly, the presence of resveratrol during the virus adsorption step was able to inhibit viral RNA synthesis by more than 60% suggesting that the molecule may also exert an effect on viral entry into cells. This hypothesis could be supported by results obtained in a molecular docking study that revealed highly stable bound conformation of resveratrol to the viral protein: ACE2 receptor complex (51). Partially similar results were obtained in a study, published by bioRxiv (52). In Vero E6 cells virus replication, evaluated by plaque assay, was reduced respectively by 50% and 90% at resveratrol concentrations of 66 and 119 μM. Significant antiviral activity was observed up to 40 hours post-infection, a time-point roughly corresponding to 5 rounds of viral replication, demonstrating the long-lasting antiviral effect of the compound. No direct virucidal effect was showed and contrary to what was observed by Yang et al. (50) only a slight reduction in virus particle production was observed when the compound was present only during the virus adsorption step, possibly due to the different experimental conditions used in the two studies. In contrast, a significant inhibitory activity on virus replication was detected when the compound was added after removal of the virus inoculum, but not when resveratrol was added at 4 or 6 hours post-infection. Therefore, resveratrol interferes with the viral infectious cycle at an early stage of the infection, i.e., on RNA replication and structural protein transcription and translation, but before virus assembly and release occur. The antiviral activity of resveratrol was then tested in primary human bronchial epithelial cells obtained from healthy individuals and differentiated on air-liquid interface cultures (52). The cells were inoculated with SARS-CoV-2 (MOI 5) in the presence of resveratrol 150 μM and, at 48-hour post-infection, a 99.3% reduction of virus titers was detected when compared to control cultures. In a similar study, resveratrol, lopinavir/ritonavir, and chloroquine were tested for their ability to reduce replication of the human coronavirus (HCoV)-229E on MRC5 cells (53). A reduction of the viral titer with resveratrol (EC50 = 4.6 μM), lopinavir/ritonavir (EC50 = 8.8 μM) and chloroquine (EC50 = 5 μM) was observed. Resveratrol was less cytotoxic (50% cytotoxic concentration (CC50) = 210 μM) than lopinavir/ritonavir (CC50 = 102 μM) and chloroquine (CC50 = 68 μM). Resveratrol inhibited HCoV-229E replication by 80% in MRC5 cells infected both with a high viral titer (MOI = 1) and a low viral titer (MOI = 0.01), with no significant difference in the effect when cells were treated before or after the infection. In the same study, inhibition of SARS-CoV-2 infection on Vero E6 cells was tested in the presence of resveratrol, but not of lopinavir/ritonavir or chloroquine because of the toxicity of these two molecules on that cell line (53). Resveratrol inhibited SARS-CoV-2 replication by 3 logs at 25 μM, the percentage of inhibition ranging from 0 to 99.93% when increasing the concentration of resveratrol from 0 to 25 μM with an EC90 and EC50 of 11.42 μM and 10.66 μM, respectively, further demonstrating the optimal antiviral activity with low cytotoxicity of this molecule *in vitro*. Preliminary data from the Molecular Medicine Department of the University of Padua (Italy), on ex-vivo nasal epithelia infected with SARS-CoV-2, confirm both the anti-viral activity and the low toxicity of resveratrol (unpublished).

## Resveratrol Activities on Pathogenetic Mechanisms Involved in COVID-19 Severity

**Figure 2:**
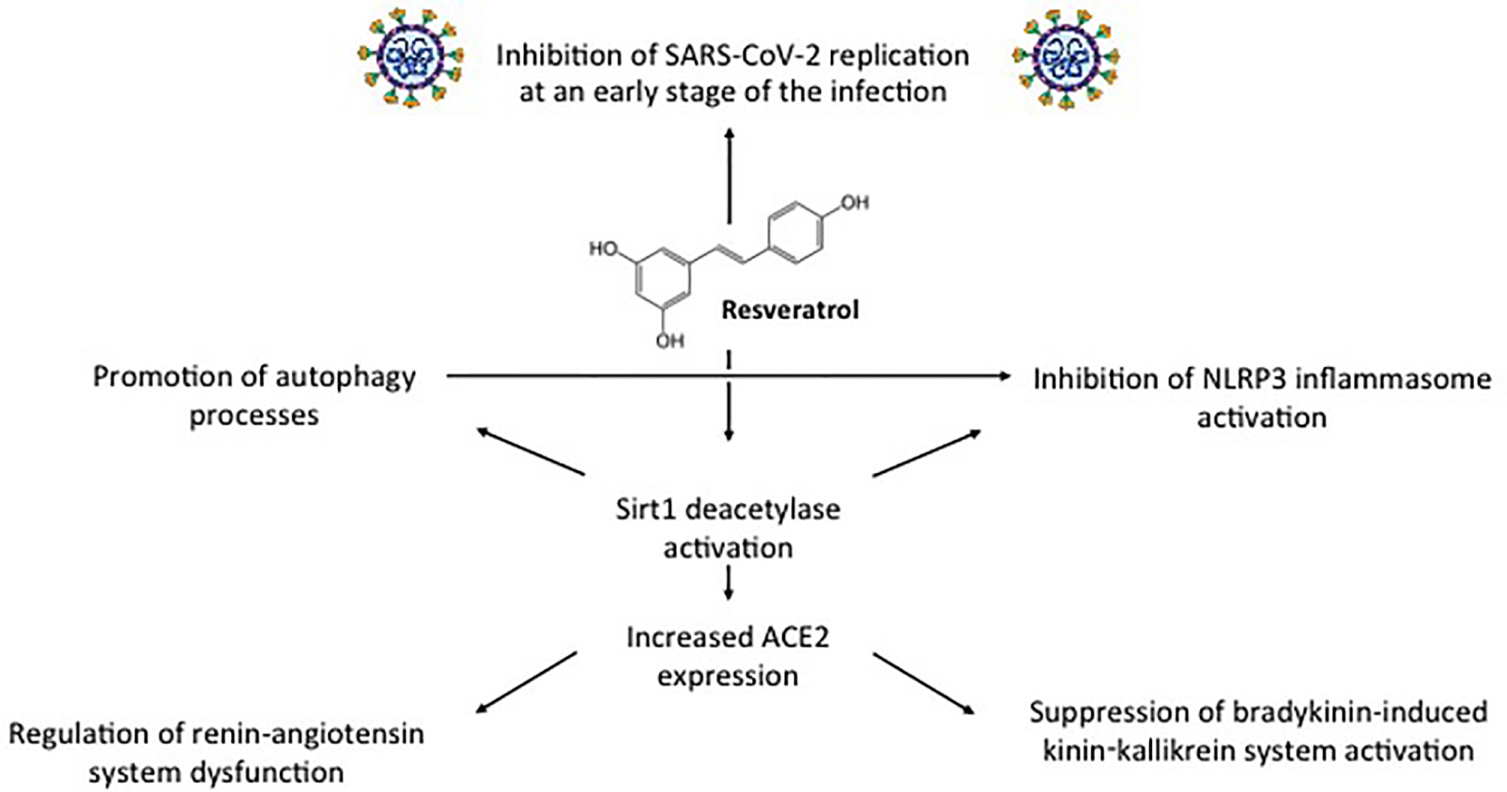
Direct antiviral activity against SARS-CoV-2 and other inhibitory functions displayed by resveratrol through Sirt1-ACE2 activation on the pathogenetic mechanism involved in COVID-19 severity.

In addition to exhibiting direct antiviral activity, resveratrol displays inhibitory functions on the pathogenetic mechanism involved in COVID-19 severity (**Figure 2**) (11). These include dysregulated NLRP3 inflammasome activation, renin-angiotensin system dysfunction, and kinin−kallikrein system stimulation. As we will see in the next paragraphs, these inhibitory functions are mediated by the induction of Sirt1 protein and of Sirt1-induced upregulation of ACE2 protein expression (54).

### NLRP3 Inflammasome, Autophagy, and SIRT1 Activation

**Figure 3:**
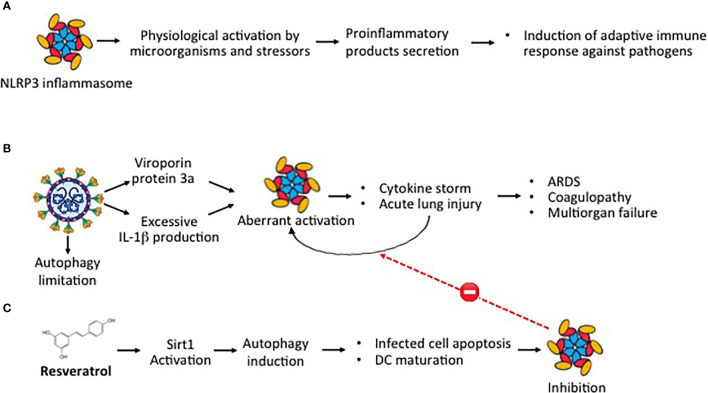
SARS-CoV-2 and aberrant NLRP3 inflammasome activation **(A)** Physiological activation of NLRP3 inflammasome by microorganisms and stressors induce the secretion of proinflammatory products with induction of adaptive immune response against pathogens. **(B)** Aberrant dysregulated activation of NLRP3 inflammasome by the SARS-CoV-2 viroporin protein 3a and excessive IL-1β release promotes cytokine storm and acute lung injury, leading to the most severe Covid-19 complications. **(C)** Through Sirt1 activation, resveratrol induces autophagy, a cellular function that facilitating self-digestion of misfolded or unused protein and cellular debris, facilitates infected cell apoptosis and dendritic cell maturation, downregulating the aberrant dysregulated NLRP3 activation.

NLRP3 inflammasome is a multiprotein complex that exists as a latent monomer in quiescent cells. The physiological activation of NLRP3 inflammasome by stressors or pathogenic microorganisms facilitates the processing of the pro-inflammatory cytokines and promotes the induction of adaptive immune response against bacteria and viruses, but its aberrant dysregulate activation is involved in the pathogenesis of several inflammatory disorders (**Figure 3A**) (55, 56). SARS-CoV-2 can directly activate NLRP3 inflammasome with a viral protein, named viroporin protein 3a, and indirectly through excessive production of IL-1β, a recognized causative factor for the more severe COVID-19 complications (**Figure 3B**) (57–59). NLRP3 inflammasome activation is further amplified by the ability of SARS-CoV-2 infection to limit autophagy, increasing the degradation of the autophagy initiating protein Beclin-1 (60). Autophagy can control NLRP3 inflammasome activation through the regulatory interactions with a variety of immune signaling pathways and the removal of endogenous inflammasome agonists (61). As demonstrated in RSV infection, autophagy not only promoted infected epithelial cell apoptosis and self-digestion of cellular debris but also facilitated dendritic cell maturation and viral antigen presentation to T-cells, thus decreasing the ineffective inflammatory response (35). A correlation between the induction of autophagy and reduction of NLRP3 inflammasome activity was demonstrated in the lung tissue of mice infected by the influenza A H1N1 virus (62). Resveratrol can activate Sirt1, a deacetylase exerting protective effects on a variety of cellular functions, including apoptosis (**Figure 3C**) (63, 64). In the lung of RSV-infected mice, SIRT1 promoted autophagy-mediated processes leading to dendritic cell activation, viral antigen presentation to T-cells, and effective antiviral immune response (65). Thus, through the downregulation of the excessive inflammatory response, shown in other viral infections (29, 32–34), and the upregulation of Sirt1 activity, resveratrol can inhibit NLRP3 inflammasome activation, inducing autophagy (66). Resveratrol-induced upregulation of SIRT1 activity, autophagy promotion, and inhibition of NLRP3 expression have been reported also in mouse models of sepsis-associated encephalopathy and in a spinal cord contusion rat model (67–69).

### ACE2 and the Renin-Angiotensin System Function

**Figure 4:**
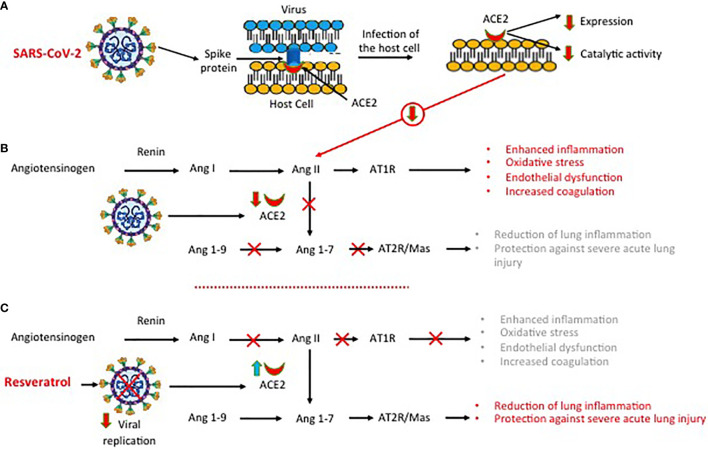
SARS-CoV-2, angiotensin-converting enzyme 2 (ACE2) expression and dysregulation of the renin-angiotensin system. **(A)** Infection of the host cell by SARS-COV-2 is associated with a down-regulation of ACE2 protein expression and function. **(B)** The downregulation of ACE2 protein function inhibits the conversion of angiotensin II (Ang II) into Ang 1-9 and then to Ang 1-7, leading to dysfunction of the renin angiotensin system with excessive production of pro-inflammatory and pro-oxidant agents. **(C)** Inhibiting SARS-CoV-2 replication, resveratrol reduces lung inflammation and lung injury.

In COVID-19, the expression and the catalytic activities of ACE2 protein are down-regulated after the SARS-CoV-2 entry into the host cells (**Figure 4A**) (49, 70). ACE, a component of the renin-angiotensin system, is a zinc metalloprotease that catalyzes the cleavage of the C-terminal dipeptide from Ang I (71). This down-regulation negatively affects some positive functions of ACE2 protein, including the conversion of angiotensin II (Ang II) into Ang 1-9 and then to Ang 1-7 (71, 72). Inhibition of Ang II conversion may lead to dysfunction of the renin-angiotensin system (RAS) and to excessive production of pro-inflammatory and pro-oxidant agents, causing acute lung injury and alveolar edema (**Figure 4B**), as shown in animal model studies (72, 73). In wild-type C57Bl/6 mice infected with SARS-CoV, the reduction of ACE2 expression was associated with a significant increase in Ang II levels and pathological changes in the lung tissue (74). Inhibition of Ang II conversion through blockage of its type 1 receptor (AT1R) attenuated the severe damages to the respiratory structures. Similarly, in different mice studies, it was shown that the upregulation of ACE2 protein and of Ang II receptor (AT1R) protected mice from bleomycin-induced lung fibrosis (75) and from severe acute lung injury, induced by acid aspiration or sepsis (76). Inhibiting SARS-CoV-2 replication, resveratrol may restore the physiological RAS function (**Figure 4C**).

### ACE2 and the Kinin−Kallikrein System Function

ACE2 can also act on the kinin−kallikrein (KK) system through the production of active peptides such as bradykinin (BK), Lys-BK, [des-Arg9]-BK (DEABK), and Lys-[des-Arg9]-BK (LDEABK) (77). Stimulating the secretion of tissue plasminogen, BK plays an important role in thrombus formation, whilst DEABK and LDEABK cause the release of proinflammatory cytokines from airway epithelial cultures and promote neutrophil infiltration and lung injury (78). ACE2, which does not inactivate BK, can cleave the terminal residue in DEABK and LDEABK, rendering these BK metabolites unable to interact with their BRB1 receptor. In SARS-CoV-2 infection, the reduced expression of ACE2 protein generates an imbalance in the KK system causing an overactivation of the DEABK/LDEABK/BRB1 axis. Dysregulation of the KK system will contribute to increased inflammation and will render the lung environment more prone to local vascular leakage and angioedema (79). When plasma leakage occurs due to tissue damage, kallikrein will be activated locally resulting in DEABK and LDEABK production and further B1BR stimulation.

### Increased ACE2 Expression and Modulation of the RAS and KKS Systems

Since a major component of COVID-19 pathogenesis is the loss of the ACE2 protective effects, the upregulation of its expression can be an attractive treatment strategy (80). An increase in ACE2 expression is not harmful to the patient as shown by the demonstration that ACE inhibitors, which increase ACE2 expression, do not increase the susceptibility to COVID-19 nor SARS-Cov-2 host cell entry and propagation (81, 82). The ACE2 upregulation-dependent beneficial effect of resveratrol on RAS dysfunction was demonstrated in animal models of aging kidneys and of abdominal aortic aneurysm. The Ang II/AT1R axis plays an important role in the aging process of the kidney, through a progressively increasing oxidative stress, whilst the angiotensin AT2R/Ang 1-7/MasR axis, which counteracts the effects of Ang II, is protective for end-organ damage. The ability of resveratrol (RSV) to modulate RAS in aging kidneys was evaluated, in eighteen-month-old male C57BL/6 mice (83). Treatment with resveratrol (40 mg/kg chow) was associated with better renal function, reduced albuminuria, and improved renal histologic findings. ACE2 expression and renal and serum Ang 1-7 levels were significantly increased in the resveratrol-treated group, as compared to the control group, with the enhancement of the AT2R/Ang 1-7/MasR axis and suppression of the Ang II/AT1R axis. Additionally, the expression of NADPH oxidase (a major product of DNA oxidation), of collagen IV, and of fibronectin was reduced, while that of endothelial nitric oxide synthase and superoxide dismutase 2 was increased (83). Thus, resveratrol exerts protective effects on aging kidneys through ACE2 Ang II suppression and MasR activation. The effect of ACE2 on abdominal aortic aneurysm development was evaluated in apolipoprotein-deficient mice^ApoE-/-^, animals prone to develop atherosclerosis (84). In these animals, resveratrol treatment (0.5-g chow) was associated with reduction of aorta dilatation, decreased expression inflammation and proteolytic enzyme markers, stimulation of the ACE2-AT2R/Ang 1-7/MasR axis, and elevation of serum and suprarenal aorta tissue ACE2 and Sirt1 levels. The effect of resveratrol was also evaluated in human aortic smooth muscle cells (AoSMC) (84). Incubation of resting human AoSMC cell cultures with resveratrol for 24 hours resulted in a significant increase in ACE2 and Sirt1 gene expression and protein activity (84). Sirt1gene silencing, using small interfering (si)RNA, downregulated the positive effects on the ACE2 gene, demonstrating that the resveratrol-induced ACE2 upregulation was Sirt1-dependent. There are no data on the effect of resveratrol on SIRT1 and ACE2 expression/activity in cells infected by SARS-CoV2 nor on the activity of resveratrol on bradykinin-induced activation of the KK system. However, it was demonstrated that resveratrol, through upregulation of Sirt1 activity in rheumatoid arthritis synovial fibroblasts, suppressed the bradykinin-induced COX-2/PGE2 production (85). SARS-Cov-2 infection causes local and systemic inflammation mediated also by COX-2 eicosanoid products with metabolic dysfunction and tissue damage (86) Therefore, the resveratrol-induced inhibition of bradykinin-induced COX-2/PGE2 production might be a valuable adjunct anti-inflammatory activity in Covid-19 (87).

## Resveratrol Pharmacokinetics and Bioavailability

Despite its potential anti-viral effects, resveratrol cannot be used as such in clinical practice because of the poor stability in aqueous solutions and the low bioavailability (12, 15). Resveratrol exhibits high membrane permeability and, after oral administration, is efficiently absorbed but rapidly and extensively metabolized (15). In human volunteers, the absorption of a 25-mg oral dose was at least 70%, with peak plasma levels of the compound and of its metabolites of 491 ± 90 ng/ml (about 2 μM, i.e. the 8%) and a half-life of 9.2 ± 0.6 hours. However, only trace amounts of unchanged resveratrol (<5 ng/ml) could be detected, because of the extremely rapid and extensive biotransformation at intestinal and liver levels (15). Conjugation with sulfate and with glucuronate leads to the production of metabolites with very little bioactivity, further contributing to the low bioavailability (88). The concern of the relatively low chemical stability, rapid and extensive metabolism, and low plasma bioavailability of oral resveratrol could be circumvented by using other formulations and administration routes to achieve targeted and/or sustained release (89). Topical administration through inhaled formulations will allow us to achieve sufficiently high concentrations of the compound in the airways, the entry route of SARS-CoV-2. Amongst aerosolized suspensions, a nasal spray containing carboxymethylated (1,3/1,6)-β-D-glucan (CM-glucan) combined with resveratrol has been produced and is commercially available for clinical use (90). β-glucans are high molecular weight polysaccharides acting as “biological response modifiers”, as they can stimulate the immune system exerting anti-viral activities (87). The combination with CM-glucan greatly improves resveratrol stability in aqueous solutions and, acting in synergy, enhances its biological activities (90). The efficacy and safety of this nasal spray combination were tested in clinical trials performed on allergic children and found to reduce the severity and relapse of upper respiratory tract infections, disorders that often recognize a viral etiology (91, 92). In a real-life study, children with acute rhinopharyngitis and a clinical history of recurrent respiratory infections were enrolled, and resveratrol-CM-glucan combination or saline isotonic solution nasal sprays were administered immediately after an anti-infective 10-day treatment (93). The active compound was able to significantly reduce the number of days with nasal symptoms, but also medication use, medical visits (p < 0.001), and school absence. Moreover, in infants with acute respiratory tract infection, resveratrol-CM-glucan nasal administration was well tolerated and reduced the upper and lower respiratory tract symptoms (94). In addition, in rhinovirus-infected infants, TLR2 mRNA level in nasal samples was significantly increased in the actively treated group compared to the placebo group at 48 h follow-up (94). Since it has been demonstrated that TLR2 can be involved in type I IFNs antiviral response upon binding of viral antigens to this pattern recognition receptor (95), this result suggests that resveratrol-CM-glucan combination could promote antiviral defense mainly through TLR2 up-regulation.

For all respiratory viruses, including SARS-CoV-2, transmission occurs primarily *via* respiratory droplets inhalation and the first target are nasal epithelial cells (96). Therefore, inhibition of viral replication at that level could be useful in controlling the early stages of the infection thereby reducing viral spread to the lower airways and resulting in a reduced chance of infection transmission following a reduced viral load. Finally, an aerosol formulation containing the association of the two molecules which could reach the lower respiratory tract was developed (97). Mass median aerodynamic diameter of the resveratrol/CM-glucan combination was lower than that shown by resveratrol or CM-glucan alone (2.83 versus 3.28 and 2.96 µm, respectively). Moreover, the resveratrol/CM-glucan association resulted in the finest and most monodispersed particles and lower values for all particle size distribution parameters, in comparison to the two single components, demonstrating good suitability for simultaneous aerosol volatilization and for treatment of patients with lower respiratory tract diseases (97).

## Conclusion

Although there are no data on the use of resveratrol in patients with SARS-CoV-2 infection, the results of the studies reported above on other respiratory viruses suggest that this compound may be an adjunctive antiviral agent to consider also in Covid-19. The inhibition of SARS-CoV-2 replication in human primary bronchial epithelial cell cultures and the direct or indirect inhibitory functions on the mechanism involved in the pathogenetic of Covid-19 severity support this working hypothesis. Since the CM-glucan-resveratrol nasal spray combination is safe and readily commercially available, and the aerosol formulation containing the association of the two molecules has been developed, randomized double-blind controlled clinical trials must rapidly be conducted to prove whether these drugs could be indeed advantageous for COVID-19 treatment in the early phase of the infection. Using the updated information on the COVID-19 epidemics and the recent/new data on the experimental studies, the protocol of a clinical trial to be submitted to the Ethics Committees is being defined.

## Author Contributions

GR and PM conceived the topic concept, wrote the first draft and revised the final manuscript. OS and AC contributed to manuscript preparation and editing. All authors contributed to the article and approved the submitted version.

## Conflict of Interest

The authors declare that the research was conducted in the absence of any commercial or financial relationships that could be construed as a potential conflict of interest.
